# Pedal Amputation for Metatarsal Osteomyelitis Following a Neglected Open Salter-Harris Type 1 Metatarsal Fracture: A Case Report

**DOI:** 10.7759/cureus.92192

**Published:** 2025-09-13

**Authors:** Stéphane Kohpe Kapseu, Camille Harold Esseme Ndjie

**Affiliations:** 1 Department of Surgery, Université des Montagnes, Bangangté, CMR; 2 Department of Surgery and Surgical Specialties, Training and Research Unit in Health Sciences, Université Joseph Ki Zerbo, Ouagadougou, BFA

**Keywords:** amputation, metatarsal osteotomy, neglected fracture, osteomyelitis, pediatric

## Abstract

Metatarsal osteomyelitis in children is a rare condition. Its management is a challenge in developing countries. The aim of this case report is to present the diagnostic and therapeutic challenge of a case of osteomyelitis on a neglected open Salter-Harris type 1 fracture of the first metatarsal in a small child, which occurred after treatment by a bone setter. This is a five-year-old child who presented to our clinic with an exposed piece of bone on the medial border of the left forefoot for two months. The latter presented a functional impotence of the left lower limb related to pain. Osteomyelitis over a neglected Salter-Harris type 1 fracture of the first metatarsal was the diagnostic hypothesis retained on the basis of clinical examination and foot radiography. After removal of the sequestrum, curettage, and cleaning of the residual cavity under sedation, regular dressings allowed the wound to heal. The patient received antibiotic therapy. Pathological findings were consistent with inflammatory and necrotic bone tissue, with no evidence of malignancy. Evolution was good, with healing of the skin wound and recovery of walking. The prognosis after five months of clinical follow-up was favorable.

## Introduction

Epiphyseal plate fractures, or physis, are common musculoskeletal injuries in children with open growth plates. The management of these fractures can sometimes represent a diagnostic and therapeutic challenge [1]. In patients aged five and under, a fall from a height is the primary mechanism. Meanwhile, in patients aged over five, most fractures occur in sports facilities and are caused by a fall onto a flat surface. The metatarsal most frequently fractured in young children was the first, while the metatarsal most frequently fractured in older children was the fifth [2]. Metatarsal osteomyelitis in children is a rare condition [3]. Its management is a challenge in developing countries [4]. Limb amputations in children mainly involve the fingers and then the toes and are generally linked to high-energy trauma [5]. While pediatric pedal amputation for osteomyelitis is rare, it is frequently reported in adult diabetic patients [6]. In resource-limited settings, certain rare pediatric nosological entities, such as neglected open fracture with epiphyseal detachment of a metatarsal, may be encountered. The aim of this case report is to present the diagnostic and therapeutic challenge of a case of osteomyelitis on a neglected open Salter-Harris type 1 fracture of the first metatarsal in a small child, which occurred after treatment by a bone setter.

## Case presentation

**Figure 1:**
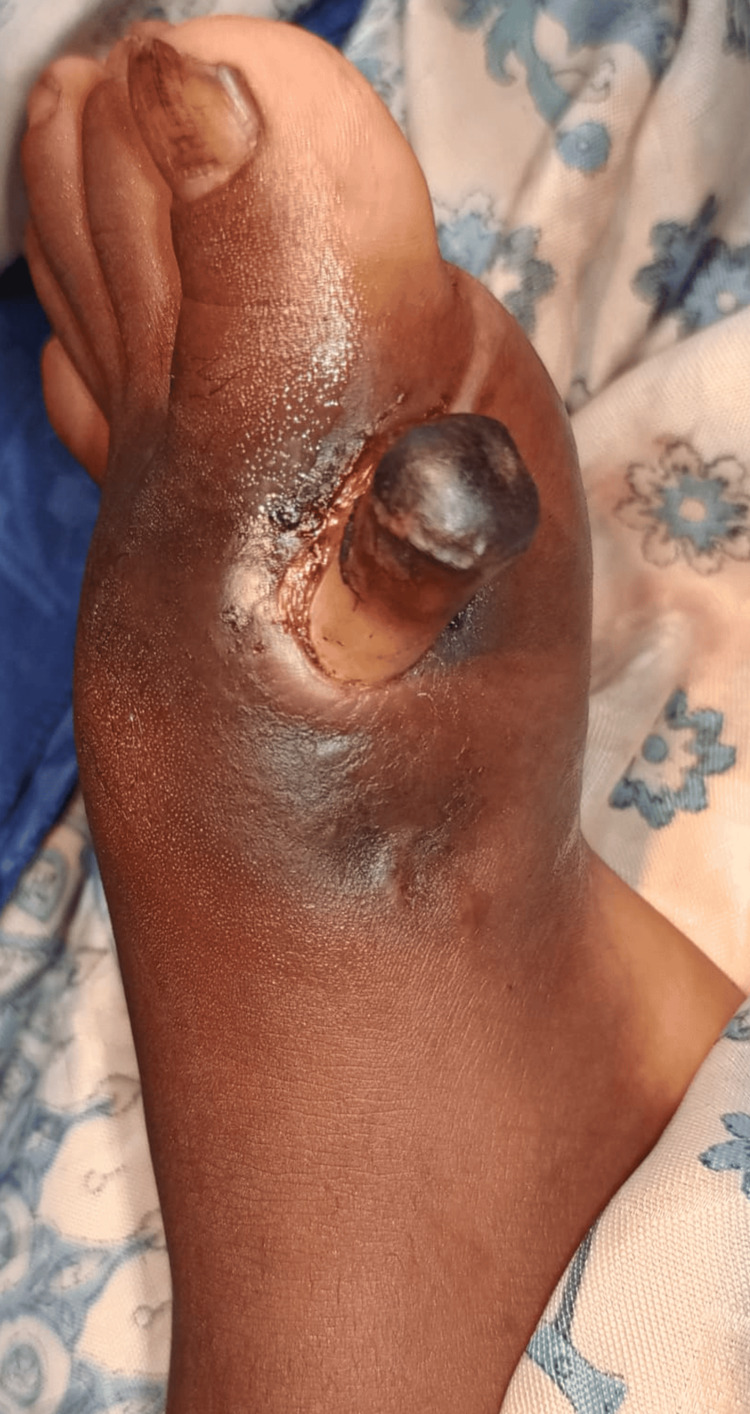
Condition at admission Exposed bone (black star)

We present a five-year-old boy who presented to our clinic with an exposed piece of bone on the medial border of the left forefoot for two months (Figure 1). The latter presented a functional impotence of the left lower limb related to pain. The patient lives in a rural area and has a precarious socioeconomic status. He has no notable medical history, has received all his vaccinations, and was born without congenital malformation. There was no history of trauma, use of vitamin D and calcium supplementation, or previous history of fractures and dislocations. He has benefited from traditional massages, which have led to his current clinical condition.

General condition was fair, vital parameters are correct, temperature is normal, and BMI is 21 kg/m^2^. The left foot is warm, painful, and enlarged compared with the contralateral side. Dorsal and plantar flexion of the foot was limited due to pain; edema mainly involved the forefoot, pedal, and posterior tibial pulse were perceptible. A growth is seen on the medial border of the left hallux, suggesting a fragment of necrotic bone (Figure 1). The absence of left inguinal adenopathy was noted.

**Figure 2:**
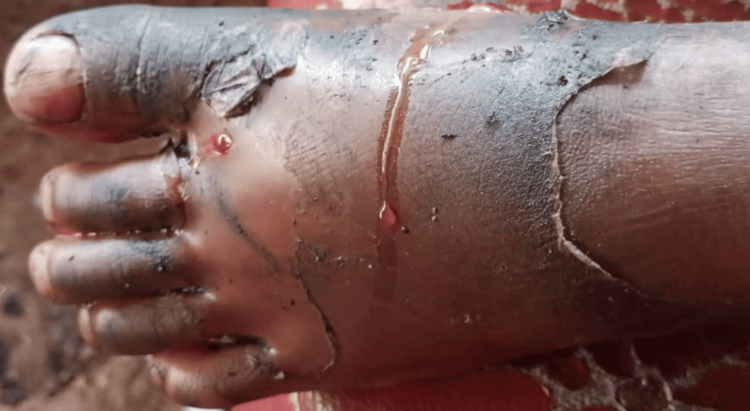
Left foot three months earlier Edema, peeling of the skin, and exudation of fluid were due to inflammation tribute to massage and local application of traditional medicines.

Three months prior to the present consultation, he had spontaneously presented with pain in his left hallux, for which his parents had opted for treatment by a bone setter. A fragment of bone was progressively exposed at the medial edge of the left hallux, and a limp on walking set in, followed by functional impotence (Figure 2).

**Figure 3:**
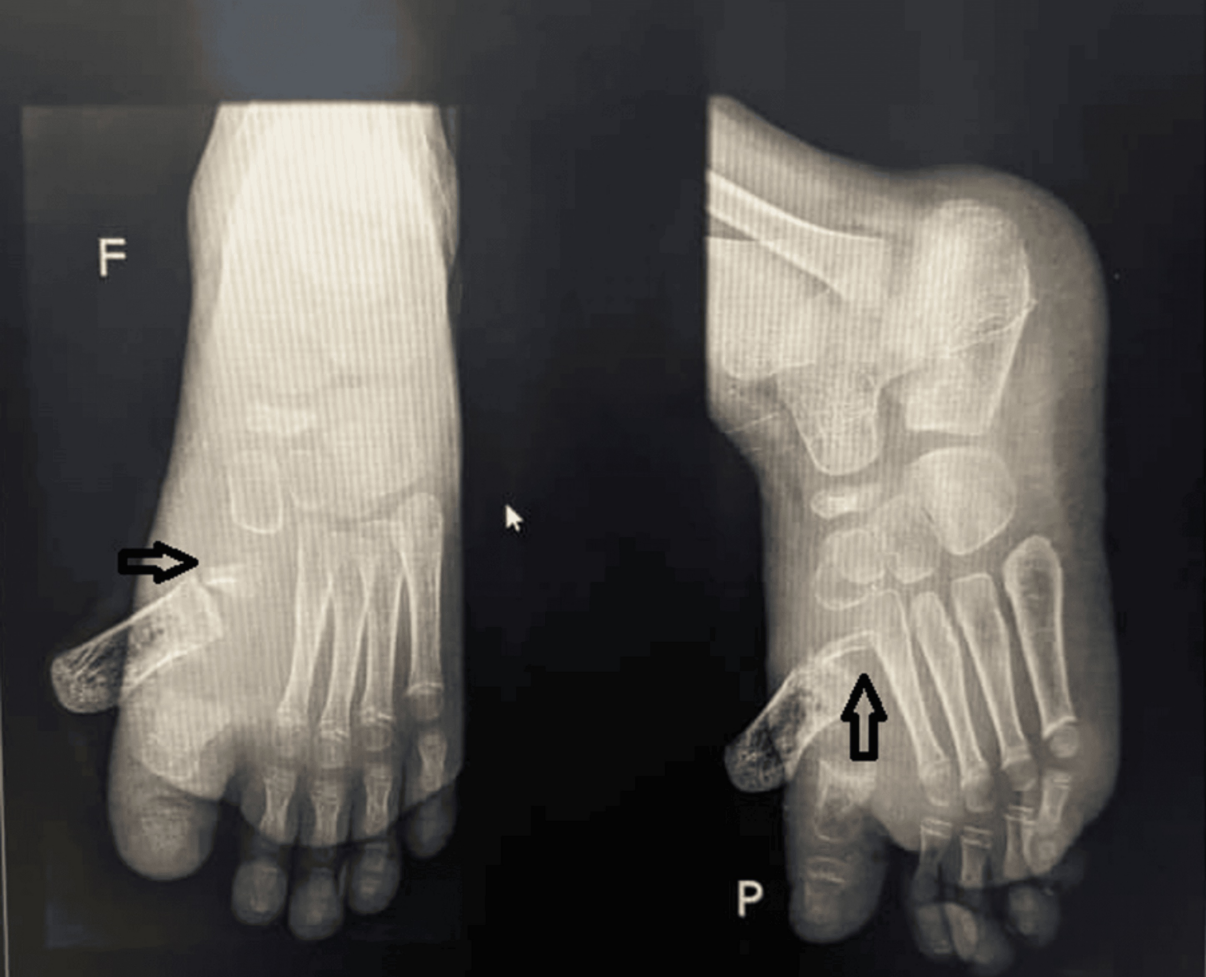
Left foot X-ray Salter-Harris type 1 fracture of the left first metatarsal (black arrow) (F: front view, P: side view)

An X-ray of the left foot revealed an open Salter-Harris type 1 fracture of the first metatarsal (Figure 3). Biological tests were consistent with infection.

The patient had no health insurance; his grandparents covered the cost of treatment. Our rural health center is a fourth-category hospital. It has no scanner or magnetic resonance imaging. Pathology examinations are outsourced.

A neglected open fracture of the left first metatarsal was suggested, but open dislocation of the left first metatarsal and metatarsal osteosarcoma were not formally ruled out. Osteomyelitis over a neglected Salter-Harris 1 fracture of the first metatarsal was the diagnostic hypothesis retained on the basis of clinical examination and foot radiography. The prognosis was considered good in view of the apparent benignity of the condition.

Povidone-iodine dressings covering the exposed bone and ulcerated skin every two days were applied prior to surgical management.

The treatment objectives were to remove the bone sequestrum and eradicate the infection. However, we were faced with two treatment options: the first was to remove the sequestrum and treat conservatively, in the knowledge that the bone loss could be filled by fibrosis. The second option was to remove the sequestrum and replace it with cement, using a “mini-masklet” technique.

**Figure 4:**
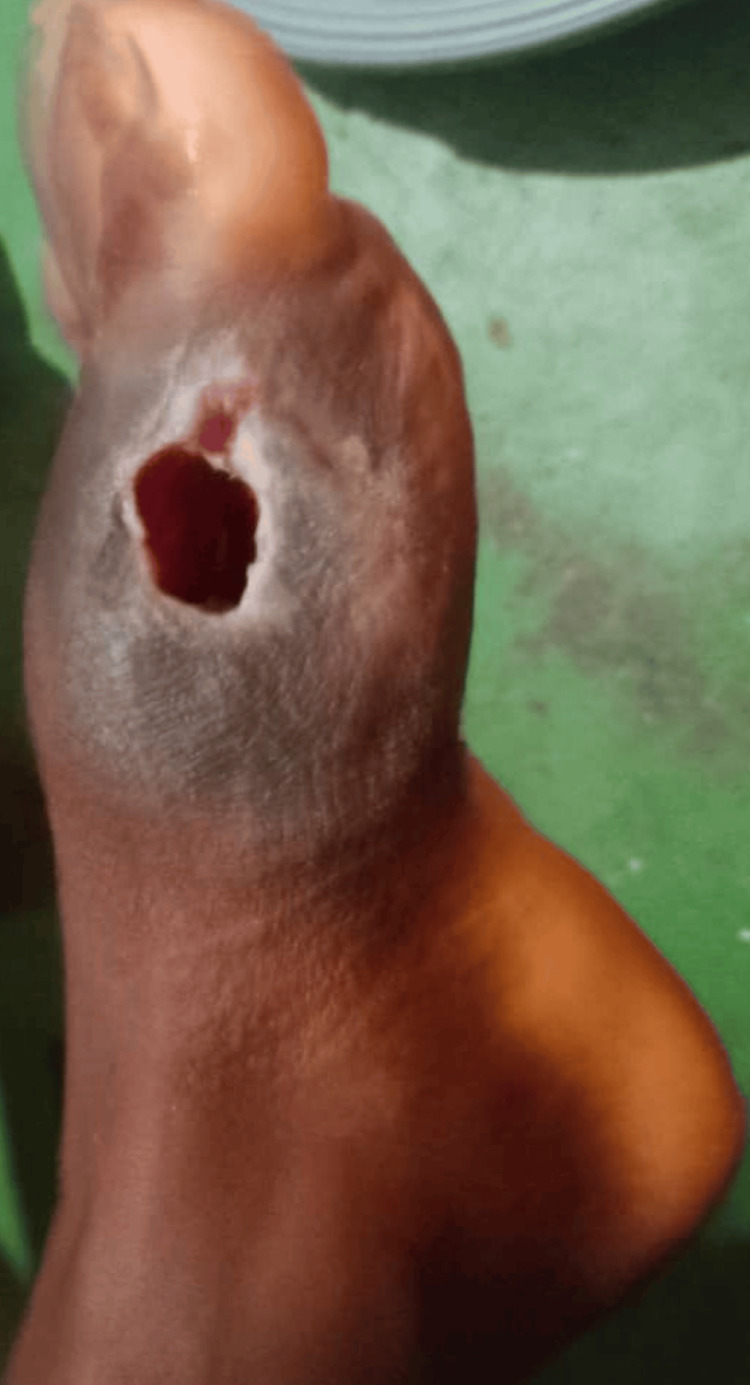
Postoperative image Image of the residual wound after removal of bone sequestrum.

We opted for the first treatment option, which appeared to be the simplest and least costly to implement. After the removal of the sequestrum, curettage, and cleaning of the residual cavity under sedation, regular dressings allowed the wound to heal (Figure 4). The patient received antibiotic therapy with amoxicillin + clavulanic acid at a dose of 100 mg/kg/day, divided into four injectable administrations per day for seven days and then three per os administrations for seven days.

**Figure 5:**
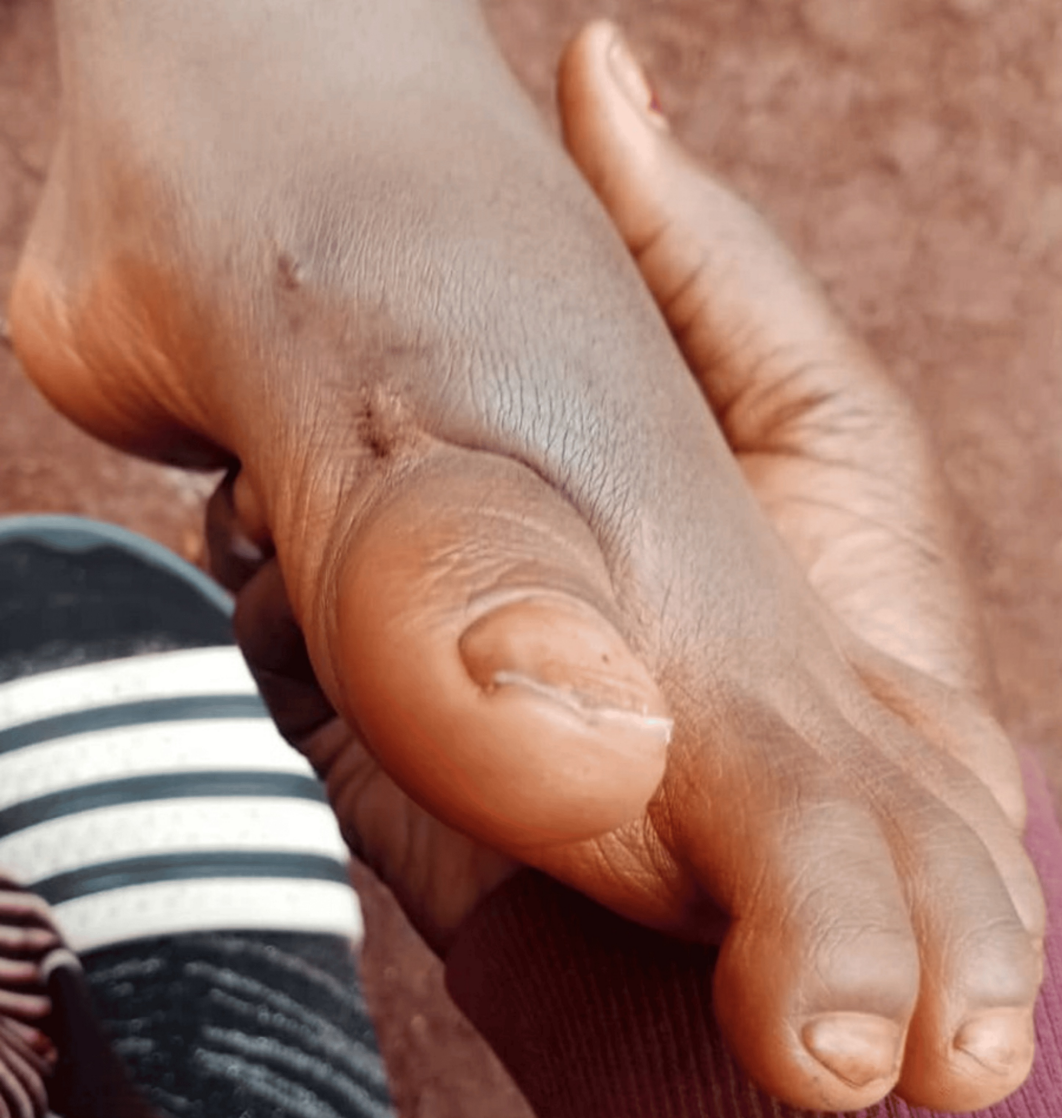
Evolution after five months Scar on the medial border of the left hallux and retraction fold due to fibrosis.

Culture of the curettage debris identified *Staphylococcus aureus *sensitive to amoxicillin + clavulanic acid. Pathological findings were consistent with inflammatory and necrotic bone tissue, with no evidence of malignancy. Evolution was good, with healing of the skin wound and recovery of walking. The prognosis after five months was favorable. However, skin retraction was observed at the base of the left hallux, probably due to fibrosis (Figure 5).

## Discussion

This observation highlights a nosological entity that is rare in the literature: neglected open Salter-Harris 1 fracture of the first metatarsal. Although limited resources are a factor to be taken into account, the diagnostic complexity of osteoarticular infections in children is not related to the technical platform [7]. Treatment options depend on the expertise of the medical team and the technical facilities available. One limitation is the lack of regular paraclinical follow-up. Indeed, in low- and middle-income countries, one of the problems faced by medical teams dealing with pediatric osteomyelitis is the lack of availability of bacterial cultures and the absence of follow-up [4]. The absence of complaints and the functionality of the limb after several months were the arguments for a good prognosis in our patient. However, skin retraction later presages shortening of the hallux length.

The aim of pedal amputation, despite the complications sometimes associated with it, is to achieve definitive healing of the foot [8]. Pediatric metatarsal sequestrectomy, which leaves a defect to be filled with fibrous tissue, may have an impact on foot function. Indeed, after internal pedal amputation, complications may be vascular, infectious, orthopedic, neurological, or psychosocial [9]; complications, which in our case were skin retraction (Figure 5) with a follow-up of several months. On the other hand, in view of the results of internal pedal amputation, which has already proved its worth in diabetic patients, it could be considered a serious therapeutic option.

The literature on the use of the Masquelet technique for osteomyelitis of the extremities is very limited in adults [10,11] and even more so in children. The “mini-masquelet” technique is a strategy for treating osteomyelitis and reconstructing bone loss after a metacarpal fracture [8].

Metatarsal sarcoma disguised as acute osteomyelitis has already been reported in adults in the literature. However, primary osteosarcoma is the most common bone malignancy in children that is not related to marrow cells, although metatarsal involvement is rare [12]. Although considered a differential diagnosis, clinical, radiological, and pathological data can be used to rectify the diagnosis [13].

## Conclusions

All pediatric osteoarticular pain must be investigated in a health facility with appropriate technical facilities. Inadequately or belatedly managed, they can compromise the functional prognosis of the affected limb. This type of clinical situation is an excellent case study to share with other pediatric orthopedic experts.
